# Role of Histone Deacetylases in Gene Regulation at Nuclear Lamina

**DOI:** 10.1371/journal.pone.0049692

**Published:** 2012-11-30

**Authors:** Beatrice C. Milon, Haibo Cheng, Mikhail V. Tselebrovsky, Sergei A. Lavrov, Valentina V. Nenasheva, Elena A. Mikhaleva, Yuri Y. Shevelyov, Dmitry I. Nurminsky

**Affiliations:** 1 Department of Biochemistry and Molecular Biology, University of Maryland School of Medicine, Baltimore, Maryland, United States of America; 2 Department of Molecular Genetics of Cell, Institute of Molecular Genetics RAS, Moscow, Russia; 3 Department of Viral and Cellular Molecular Genetics, Institute of Molecular Genetics RAS, Moscow, Russia; National Taiwan University, Taiwan

## Abstract

Theoretical models suggest that gene silencing at the nuclear periphery may involve “closing” of chromatin by transcriptional repressors, such as histone deacetylases (HDACs). Here we provide experimental evidence confirming these predictions. Histone acetylation, chromatin compactness, and gene repression in lamina-interacting multigenic chromatin domains were analyzed in Drosophila *S2* cells in which B-type lamin, diverse HDACs, and lamina-associated proteins were downregulated by dsRNA. Lamin depletion resulted in decreased compactness of the repressed multigenic domain associated with its detachment from the lamina and enhanced histone acetylation. Our data reveal the major role for HDAC1 in mediating deacetylation, chromatin compaction, and gene silencing in the multigenic domain, and an auxiliary role for HDAC3 that is required for retention of the domain at the lamina. These findings demonstrate the manifold and central involvement of class I HDACs in regulation of lamina-associated genes, illuminating a mechanism by which these enzymes can orchestrate normal and pathological development.

## Introduction

Numerous clusters of genes, co-expressed in development or in disease, have been described in diverse eukaryotes [1]–[3]. Silencing of such clusters usually involves interactions with nuclear lamina [4]–[8] while their transcriptional activation is concomitant with the loss of these interactions [5]–[7], [9], [10]. Furthermore, artificial recruitment of genes to the lamina in model systems causes silencing [11]–[13]. These observations strongly indicate that proximity of co-expressed gene-clusters to the lamina may define their repression. We have previously shown that B-type lamin, the major component of lamina, is critical in silencing of tissue-specific co-expressed gene-clusters at nuclear periphery [7] but further details of underlying mechanism(s) remained obscure. A theoretical model [14] suggests that accessory polypeptides such as Lamin B receptor (LBR) and LEM domain proteins tether diverse transcriptional repressors to the lamin meshwork thereby creating a “silencing environment”, which acts on chromatin that is ensnared at the nuclear periphery by chromatin/lamina-bridging factors such as barrier-to-autointegration factor (BAF) [14], [15]. The abundance of H3K27 and/or H3K9 histone methylation in some lamina-associated domains supports the presence of silencing factors that act at the nuclear periphery through these histone modifications [4], [5], [16], [17]. Among these, the roles for Polycomb system [18] and HP1 interacting with LBR [19] have been proposed. Silencing of H3K27me3- and H3K9me3-enriched large tandem transgene arrays at the nuclear periphery appears to be dependent on BAF and LEM domain proteins [20], indicative of their involvement in repression. However, these artificial heterochromatinized tandem arrays differ substantially from the endogenous lamina-associated gene-clusters containing mostly non-repetitive genes [1], [7]. Moreover, only a small fraction of lamina-bound genes interact with Polycomb or HP1 in *Drosophila* [7], [16], [21]. These observations imply a relatively minor role for these factors in the lamina-dependent gene silencing in flies. In contrast, histone deacetylation is commonly found in the native lamina-associated silent chromatin [6], [22] and occurs on genes artificially recruited to the lamina, concomitant with their repression [12]. A broad-range HDAC inhibitor, trichostatin A (TSA), increases histone acetylation at the nuclear periphery [23] and attenuates repression of lamina-tethered genes [13], supporting the idea that HDACs may mediate gene repression at the nuclear lamina where these enzymes can accumulate due to their affinity to the LEM domain proteins and BAF [15], [24], [25]. Given that histone deacetylation has been linked to compaction of chromatin [26]–[28], it is also conceivable that HDAC-mediated silencing at the nuclear periphery may involve “closing” of chromatin domains – a phenomenon long known as a characteristic of gene repression [29], [30]. However, specific HDAC enzyme(s) responsible for repression have not been identified and the mechanisms of repression are not completely understood. HDACs are classified into four classes including Class I of Zn^2+^-dependent nuclear enzymes, Class II HDACs that show nucleocytoplasmic localization, NAD^+^-dependent enzymes of Class III, and Zn^2+^-dependent Class IV (see [31] for overview). Six histone deacetylases have been identified in *Drosophila melanogaster*: HDAC1 and HDAC3 of Class I, HDAC2 and HDAC4 of Class II, dSIR2 of Class III and HDACX of Class IV [32], [33]. Among these enzymes, HDAC1 and 3 play a major role in the regulation of gene expression [32]. By taking advantage of the well-described model of testis-specific multigenic chromatin domain repressed by a nuclear lamina-dependent mechanism in *Drosophila* somatic cells [1], [7], [34], we herein show that this repression is linked to histone deacetylation and chromatin condensation. We have also performed a comprehensive survey of all four classes of Drosophila HDACs, and identified histone modifying enzymes responsible for these effects.

## Materials and Methods

### Cell culture and RNAi

Schneider 2 (*S2*) cells (ATCC) were maintained in Schneider's *Drosophila* Medium (Invitrogen) supplemented with 10% heat inactivated fetal bovine serum and 1% penicillin-streptomycin mixture. For HDAC inhibitor treatment, *S2* cells were incubated with 250 nM Trichostatin A or a vehicle control (DMSO) for 48 hours. To generate dsRNA, cDNA was obtained from *S2* cell RNA with the Maxima® First Strand cDNA Synthesis Kit for RT-qPCR (Fermentas), followed by PCR with gene-specific primers carrying the T7 promoter adaptor. The PCR program consisted of 5 cycles at 94°C-30 sec, 58°C-30 sec, 68°C-1 min and 30 cycles at 94°C-30 sec, 68°C-1 min (primer sequences shown in Table S1). dsRNAs were transcribed using the MEGAscript RNAi Kit (Ambion) with 1 µg PCR product as a template. 5×10^5^ cells were plated in 12-well plates and incubated with 37 nM of dsRNA in serum-free medium for one hour. Then, medium was added to restore the serum to 10% and cells were incubated for 72 h before analysis. For FISH experiments, cells were treated with dsRNA twice with the second treatment at 72 h, and analyzed at 120 h.

### Cell growth and cell death

Cells were seeded in multi-well plates and treated with dsRNA as described above. RNAi was performed in triplicate for each dsRNA. Cells were counted at the beginning and again at the end of the experiment (three days). The growth rate was calculated as follow: Growth Rate = ln(N1/N0)/T, where N1 is the number of cells at the end of the experiment and N0 is the initial number of cells, T is the incubation time in hours. Following cell counting, cell toxicity of dsRNAs was evaluated with Live/Dead® Reduced Biohazard Viability/Cytotoxicity Kit following the manufacturer's instructions. After staining with SYTO®10 (green fluorescent nucleic acid stain labeling all cells) and DEAD Red™ (cell-impermeable red fluorescent nucleic acid stain labeling only cells with compromised membranes), cells were fixed with 4% glutaraldehyde and placed on a microscope slide. For each sample, green and red cells were counted from six to eight random fields and the toxicity was evaluated as a ratio of red cells/green cells.

### qRT-PCR

RNA was extracted from *S2* cells following the protocol for TRIzol® Reagent (Invitrogen) with additional treatment by RQ1 RNase-free DNase I (Promega) followed by purification of the RNA with the RNeasy Mini Kit (QIAGEN). The RNA was reverse transcribed with the Maxima® First Strand cDNA Synthesis Kit for RT-qPCR (Fermentas) and the cDNA products were amplified by Real-Time PCR in a LightCycler® 480 II System (Roche) with LightCycler® 480 SYBR Green I Master reagent (primers are listed in Table S2).

### Western blot

Proteins were extracted from *S2* cells with RIPA buffer supplemented with a cocktail of protease inhibitors (Thermo Scientific) and lysates were sonicated until viscosity lost. Protein concentrations were measured with Pierce® BCA Protein Assay Kit (Thermo Scientific). 30 µg protein samples were separated by SDS-PAGE and transferred onto PVDF membrane. After blocking in TBS-Tween-5% milk, membranes were incubated with LamDm_o_ antibody (ADL195, Developmental Studies Hybridoma Bank) or HDAC1 antibody (ab1767, Abcam), followed by an appropriate HRP-conjugated secondary antibody (Sigma). The bands were revealed by chemiluminescence (SuperSignal* West Pico Chemiluminescent Substrate, Thermo Scientific). The membranes were then stripped with Western-Re-Probe™ Reagent (Calbiochem) and re-probed with anti-β-Actin (ab8224, Abcam) as a loading control.

### Chromatin Immunoprecipitation Assay

ChIP assays were performed using the EZ-Magna ChIP™ A kit (Millipore) following the manufacturer's instructions. Briefly, *S2* cells were fixed with 1% formaldehyde, and resuspended in cell lysis buffer. The chromatin was sheared by sonication and immunoprecipitated overnight at 4°C in the presence of protein A magnetic beads and 5 µg of either control rabbit IgG (Millipore), or Anti-Acetyl Histone H3 or H4 (Active Motif), or Anti-Histone H3 or H4 (Abcam). After several washes, the DNA/protein complexes were eluted from the beads and the proteins were digested by Proteinase K. The DNA was then purified using spin columns and used as a template for qPCR (Primers are listed in Table S3). qPCR was performed using LightCycler®480 SYBR Green (Roche) and a LightCycler®480 II. Histone acetylation was measured as the enrichment in Acetyl-H3 or H4 ChIP and normalized by the enrichment in total H3 or H4 ChIP.

### General sensitivity to DNase I

The assay was performed as previously described [34] with minor modifications. Briefly, 1×10^6^ *S2* cells were permeabilized with 0.05% NP40 and resuspended in DNase I Buffer (40 mM Tris-HCl, 0.4 mM EDTA, 10 mM MgCl_2_, 10 mM CaCl_2_, 0.1 mg/ml BSA). An aliquot of each sample was set aside and later used as untreated control. DNase I digestion was performed at 37°C for 10 minutes with 0.1 U RQ1 DNase (Promega). After digestion, DNA was purified using the DNeasy Blood & Tissue Kit (Qiagen) and used as a template for qPCR (Primers are listed in Table S3). Sensitivity to DNase I-digestion was quantified as described in [34]. We first measured the relative qPCR yield for each amplicon as the ratio of yields observed with DNaseI-treated versus untreated samples. Then, the results were adjusted to account for the differences in amplicon length. The average relative qPCR yield observed for the amplicons A37 and A39 (at positions 37 kb and 39 kb respectively in the *60D1* region, located outside the testis-specific cluster and showing high accessibility to DNase I [34]) was calculated, and relative qPCR yields observed for individual amplicons were normalized against this value to produce normalized relative yields (NRYs). As a consequence, “open” chromatin regions result in NRYs close to 1.0, while “closed” chromatin regions, more resistant to DNase I, result in significantly higher NRY values. Finally, fold changes in NRY were calculated by normalizing the values detected for the LamDm_o_ or HDAC knockdown to those observed for the control LacZ mock-knockdown.

### Fluorescence *in situ* hybridization

FISH probe for the *60D1* region was prepared by using the 35 kb cosmid k9 [7] by random primed synthesis on 0.5 µg template samples with the DIG DNA labeling kit (Roche). The probe was purified on Micro Bio-Spin 30 columns (Bio-Rad), mixed with 25 µg of salmon sperm DNA and 250 µg of yeast tRNA, fragmented by sonication to ∼500 bp, ethanol precipitated, and dissolved in 200 µL of hybridization buffer (50% formamide, 4×SSC, 100 mM Na_3_PO_4_, pH 7.0, 0,1% Tween 20). FISH procedure was performed as described in [35] with modifications. In brief, *S2* cells were washed twice in PBS supplemented with 0.1% Tween 20 (PBT), fixed with 3.7% formaldehyde in PBT for 25 min at room temperature, washed 3 times with PBT, and treated with RNase A (100 µg/mL in PBT) overnight at 6°C. Next, cells were washed 3 times in PBT and processed through three sequential 15-min incubations in solutions containing hybridization buffer and PBT in ratios of 1∶4, 1∶1, and 4∶1. After the final 15 min incubation in hybridization buffer, 50 µL of DIG-labeled probe solution was added, samples covered with mineral oil, denatured by incubation for 10 min at 80°C, and then hybridized overnight at 37°C. After hybridization, cells were washed for 10 min at 42°C in each of the following solutions: 50% formamide, 2×SSC (twice); 40% formamide, 2×SSC, H_2_O; 30% formamide, 70% PBT; 20% formamide, 80% PBT; 10% formamide, 90% PBT; PBT. Then, cells were treated with Image-IT FX (Invitrogen) for 30 min, washed in PBT and blocked in PBT supplemented with 3% natural goat serum (Millipore) for at least 3 h at room temperature. Cells were incubated with a murine monoclonal antibody against LamDm_o_ (ADL84, Developmental Studies Hybridoma Bank), guinea pig polyclonal anti-Lamin B Receptor antibody [36], sheep polyclonal anti-DIG rhodamine-conjugated antibody (Roche) and secondary anti-mouse or anti-guinea pig Alexa 488 conjugated antibody (Invitrogen). After several washes in PBT, cells were embedded in Slow-Fade Gold (Invitrogen) mounting medium.

### Image Analysis

Three-dimensional image stacks were recorded with a confocal Zeiss LSM510 Meta microscope. Images were processed and analyzed by using IMARIS 6.1.5 software (Bitplane AG). Further analysis of the images was performed using blind experimental setup. Images were thresholded to eliminate hybridization background, and positions of signals were determined. Nuclear rim colored by anti-LamDm_o_ antibody (or anti-Lamin B Receptor antibody in case of *lamDm_o_* knockdown) was manually outlined by the middle in all stacks to reconstruct the surface of the nuclei. One measuring point was positioned on the FISH signal and another one was put on the reconstructed nucleus surface in the place of its earliest intersection with progressively growing sphere of the first measuring point. The distance D between the measuring points (the shortest distance between the FISH signal and the nuclear rim) was measured for each nucleus. In parallel, the radius R of a sphere of the volume equal to the observed nucleus volume V was calculated. Data were obtained from at least two independent experiments for *LacZ* control and *HDAC1*, *HDAC3*, *HDAC1+HDAC3* and *LamDm_O_* depletions.

The positions of signals within the nuclei were analyzed using two approaches. (i) The nuclei were ranked according to the distance D between the signal and the nuclear rim, these results are shown in Table S4. The D values equal of 0.4 µM or less were interpreted as evidence for direct contact of the locus with nuclear envelope [7]. (ii) The nuclei were ranked according to the D/R ratio, these results are shown in Table S5. The fractions of signals in the peripheral 1/3 volume shell (D≤0.126R) were calculated.

### Statistical Analyses

#### DNase I sensitivity and ChIP assays

Two tailed t-test was used to analyze the pairwise difference between the experimental (Target RNAis) and control groups (LacZ RNAi) for each amplicon.

#### Gene expression studies

One tailed t-test was performed to assess the significance of Target RNAi>*LacZ* RNAi. Non-parametric Mann-Withney test was performed to assess the significance of the difference between groups: testis-specific gene-cluster *60D1* (*Crtp*, *Yu*, *Ssl*, *Pros28.1B* and *CG13581*) versus control genes (*Rp49* and *Actin5C*). p values<0.05 were considered significant.

FISH experiment: One-tailed Z-test was applied to analyze the significance of the observed differences in the proportions of FISH signals within the peripheral 0.4 µm volume shell.

## Results and Discussion

**Figure 1:**
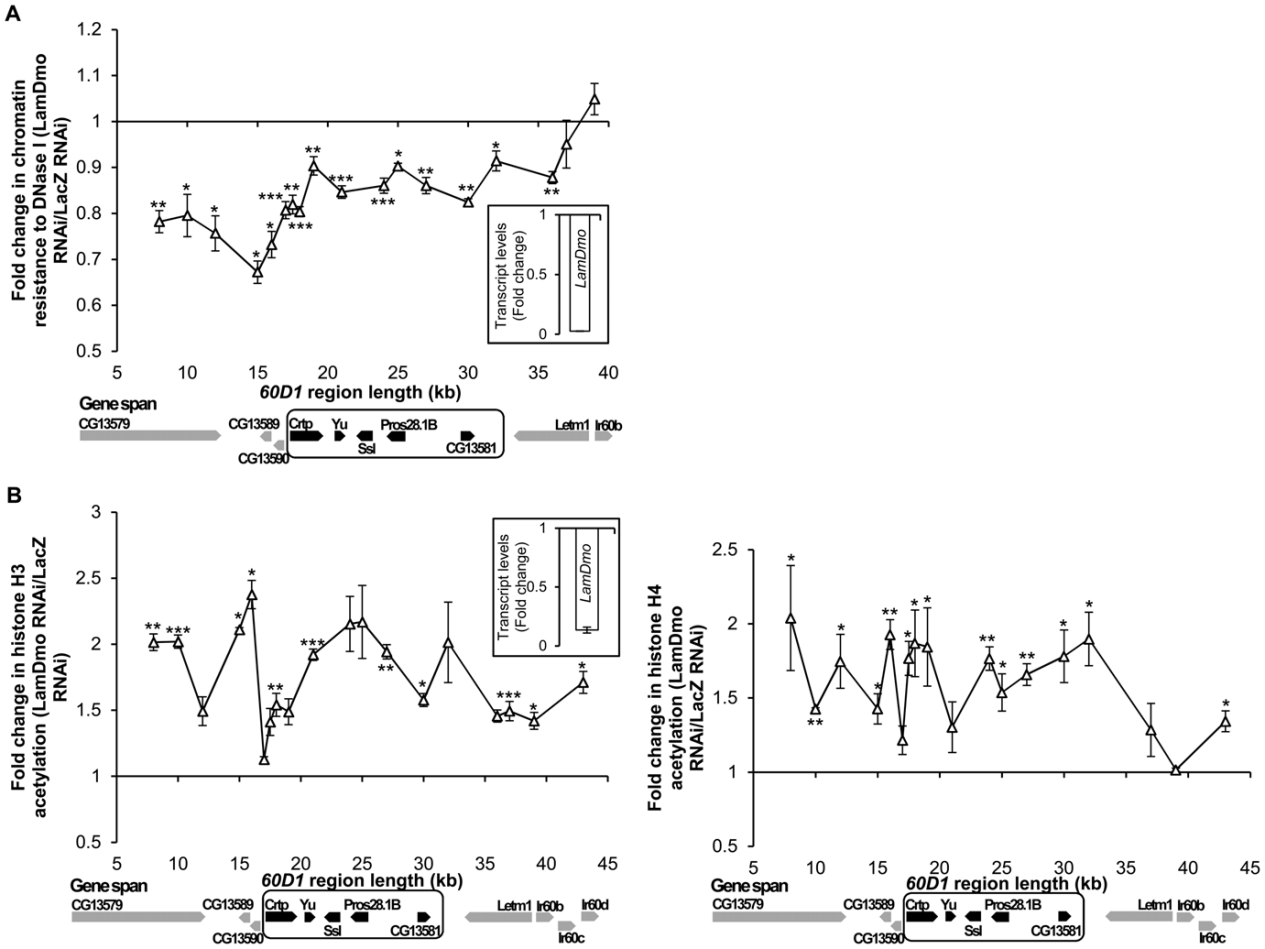
Effect of B-type Lamin depletion on chromatin compactness and histone acetylation. (**A**) Increase in general sensitivity to DNase I upon dsRNA-induced depletion of *LamDm_o_*. Permeabilized cells were treated with DNase I and DNA damage was quantified by qPCR and normalized to the amplicons located at 37–39 kb (outside the *60D1* cluster), as shown for the *LamDm_o_*-depleted cells in comparison to control *LacZ* dsRNA-treated cells. Horizontal axis shows positions of amplicons relative to the testis-specific *60D1* gene cluster outlined with a box, and its genes highlighted in black. (**B**) Increase in histone acetylation along the *60D1* cluster in *LamDm_o_* dsRNA-treated cells as compared to control *LacZ* dsRNA-treated cells. Acetylation of histones H3 (left panel) and H4 (right panel) was detected by ChIP assay. Horizontal axis is same as in (A). n = 3 to 6; error bars show SEM; *, p≤0.05; **, p≤0.01 for comparisons to the control. Inserts show the knockdown efficiency of the dsRNA at the RNA levels.

To clarify the links between nuclear architecture, local chromatin structure, and coordinate regulation of multiple genes via histone acetylation, we took advantage of a well-described model of testis-specific multigenic chromatin domain *60D1* that is repressed in *Drosophila* somatic cells by a nuclear lamina-dependent mechanism [1], [7], [34]. This domain frequently contacts nuclear lamina [7], [8] and is hypoacetylated in *S2* cells according to high-throughput analysis [37]. Downregulation of B-type lamin *LamDm_o_* in *S2* cells results in derepression of the *60D1* gene-cluster [7]. Here we analyzed the effect of dsRNA-induced knockdown of *LamDm_o_* on chromatin compactness along the *60D1* cluster, by assaying for general chromatin sensitivity to DNase I [38], [39]. The RNAi treatment resulted in 95% reduction of *LamDm_o_* transcript (Figure 1A) and ablation of LamDm_o_ protein (Figure S1A) without causing significant reduction in cell proliferation or conferring excessive cell mortality (Figure S2A, B). In our pilot studies, we found that in untreated cells the difference in chromatin compactness between the *60D1* region (“closed”) and *Actin5C* locus (“open”) can be detected over a broad range of DNase I concentrations, assuring that the assay is robust and reliable (Figure S3A, B). Further, in cells with depleted *LamDm_o_* we found a decrease in chromatin resistance to DNase I along the *60D1* cluster, indicative of reduced chromatin compactness (Figure 1A), whereas no decrease in resistance was detected in two “open” loci, *Actin5C* and *RpL9* (Figure S3C). The detected decrease in resistance to DNase I induced by *LamDm_o_* depletion varied between 10% and 35% in the *60D1* region. The maximal level of decrease is approaching the 40% difference between the *60D1* region and the actively expressed *Actin5C* locus observed under the same assay conditions (0.1 U DNase I/10^6^ cells) in untreated cells (Figure S3B). Therefore, integrity of the nuclear lamina determines “closed” inactive configuration of the multigenic chromatin domain repressed by lamina interactions, as depletion of lamin causes at least partial chromatin “opening”. The role for histone deacetylation in this repression is strongly suggested by the finding that acetylation of histones H3 and H4, detected by ChIP with antibodies recognizing the major acetylated residues of H3 (K9, K14, K18, K23,K27) and H4 (K5, K8, K12, K16) [40], is significantly increased throughout the *60D1* cluster in lamin-depleted *S2* cells (Figure 1B). The role for HDAC activity in lamina-dependent repression is further confirmed by a significant activation of the *60D1* gene-cluster in *S2* cells treated with a broad-range HDAC inhibitor TSA (Figure S4). Cumulatively, these findings support the model in which HDAC(s) silences the lamina-tethered *60D1* cluster by deacetylating histones across the repressed chromatin domain.

**Figure 2:**
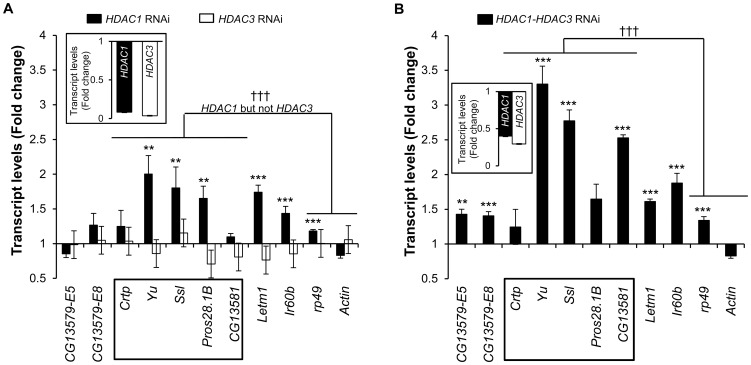
Effect of Class I HDAC depletion on expression of the *60D1* gene cluster. (**A**) Treatment of cells with *HDAC1* dsRNA results in increased transcript levels for the testis-specific cluster. Bars show changes in transcript levels detected with RT-qPCR in cells treated with *HDAC1* dsRNA or *HDAC3* dsRNA, as compared to the *LacZ* dsRNA-treated control cells. (**B**) Increased changes in transcript levels upon treatment of the cells with the mixture of *HDAC1* and *HDAC3* dsRNAs. Gene symbols are shown on the X-axis; the *60D1* gene-cluster is framed. *Rp49* and *Actin5C* are housekeeping genes used as controls. *Rpl9* served as a template for loading reference. n = 6 to 9; error bars show SEM; **, p≤0.01; ***, p≤0.001 for comparison of individual transcript levels between *LacZ* RNAi and target RNAi; †††, p≤0.001 for comparison between the *60D1* cluster and control housekeeping genes. Inserts show the knockdown efficiency of the RNAi at the RNA levels.

Next, we downregulated each of the six *Drosophila* HDACs expressed in *S2* cells using gene-specific dsRNAs (Figures 2A and S5), and monitored the effect of these treatments on expression of the *60D1* cluster. We found that downregulation of the Class II enzymes HDAC2 and HDAC4, Class III enzyme dSIR2, and Class IV enzyme HDACX did not bring significant changes to expression of the *60D1* gene cluster (Figure S5), while downregulation of the Class I enzyme HDAC1 did cause a significant (p<0.001) increase in expression of the *60D1* cluster as compared to the control housekeeping genes (Figure 2A) demonstrating the major role for this enzyme in repression. Another Class I enzyme HDAC3 does not appear essential for the repression because its downregulation did not affect transcript levels of the *60D1* gene-cluster (Figure 2A). However, this enzyme may have an auxiliary role since the double knockdown of HDAC1 (60% efficient) and HDAC3 led to a further 2-fold increase in the *60D1* transcripts as compared to the knockdown of HDAC1 alone (90% efficient) (Figure 2B). Of note, depletion of HDAC3 did not alter cell proliferation and mortality, and ablation of HDAC1 either alone or in combination with HDAC3 had only a minor effect on these parameters slowing cell growth by 20–30% and increasing the proportion of dead cells from 8.7% to 13.3% (Figure S2).

**Figure 3:**
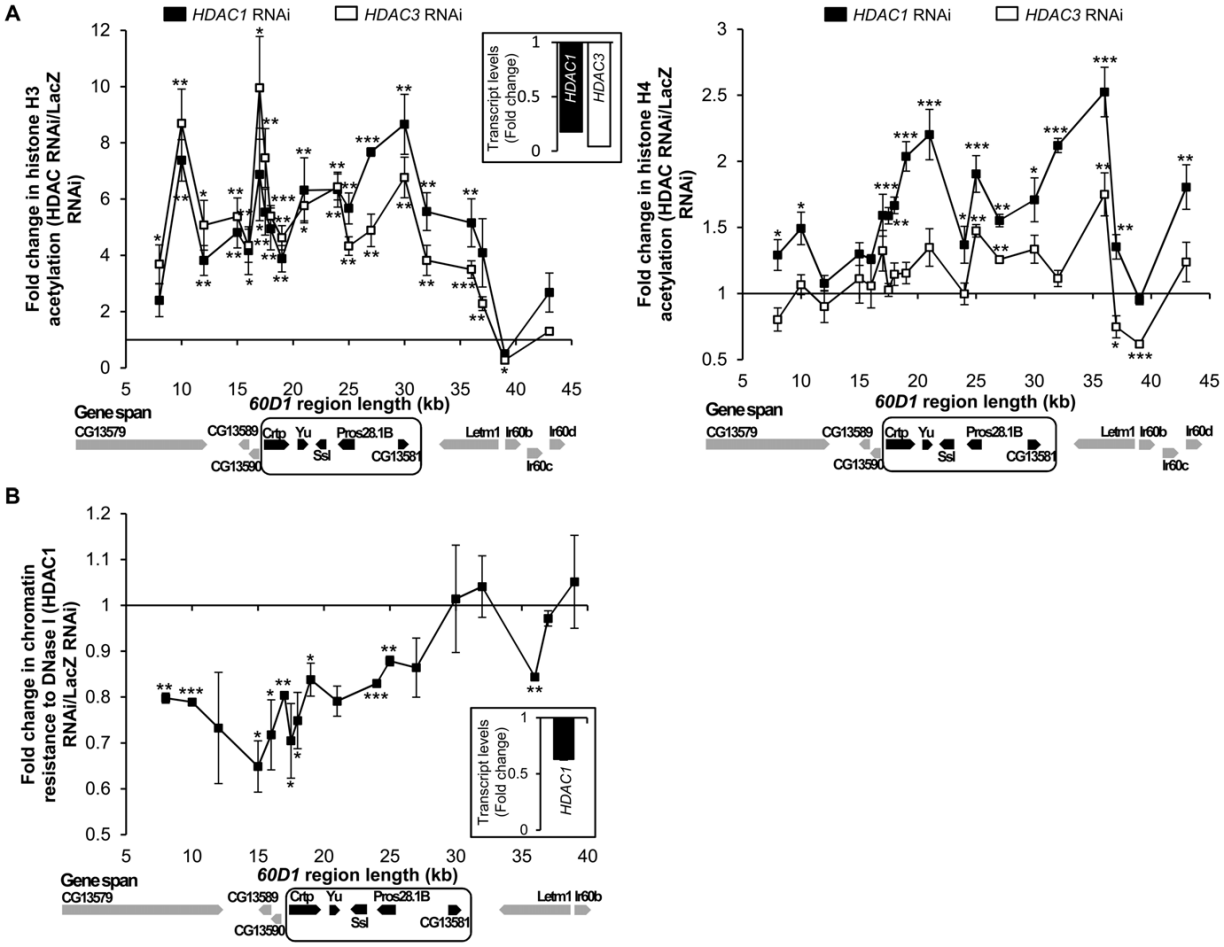
Effects of Class I HDAC depletion on histone acetylation and chromatin compactness. (**A**) ChIP assay shows increased acetylation of histones H3 (left panel) and H4 (right panel) in cells treated with *HDAC1* dsRNA and *HDAC3* dsRNAs as compared to the *LacZ* dsRNA-treated control cells. n = 4; error bars represent SEM. (**B**) Decreased chromatin compactness revealed by the general sensitivity to DNase I assay in *HDAC1* dsRNA-treated cells as compared to the control *LacZ* dsRNA treatment. Gene positions are shown below the X-axis with the *60D1* cluster framed. n = 2 to 4; error bars show SEM. *, p≤0.05; **, p≤0.01; ***, p≤0.001 for comparisons to the control. Inserts show the knockdown efficiency of the RNAi at the RNA levels.

While up-regulation of the *60D1* cluster caused by depletion of HDAC1 or HDAC1/HDAC3 was specific and significant in comparison to two randomly chosen housekeeping genes, it did spread beyond the cluster and included the adjacent genes *letm-1* and *Ir60b* that are not in tight contact with lamina [8]. This result may be explained by the fact that *HDAC1* RNAi depletes the total HDAC1 pool in the cell and therefore affects gene expression more broadly than *LamDm_o_* RNAi which would functionally disrupt only the nuclear periphery fraction of HDAC1. Nevertheless, our observation indicates involvement of HDACs 1 and 3 in gene silencing at nuclear lamina. Next, we examined the role of these enzymes in histone deacetylation and compactness of chromatin associated with the lamin-dependent repression of the *60D1* cluster. The dsRNA-mediated downregulation of HDAC1 led to hyperacetylation of histones H3 and H4 (Figure 3A) and to a significant increase in DNAse I accessibility (Figure 3B) across the entire *60D1* region. Interestingly, downregulation of HDAC3 resulted in a substantial increase in acetylation of histone H3 similarly to ablation of HDAC1, but did not cause broad and striking changes in acetylation of H4. This implies a selective requirement for specific HDACs in histone type-specific deacetylation. In this context, the fact that HDAC1 is required for efficient repression of the *60D1* cluster while HDAC3 is not, suggests that histone H3 deacetylation alone is not sufficient for gene repression and highlights the importance of histone H4 acetylation status in gene silencing at nuclear lamina.

**Figure 4:**
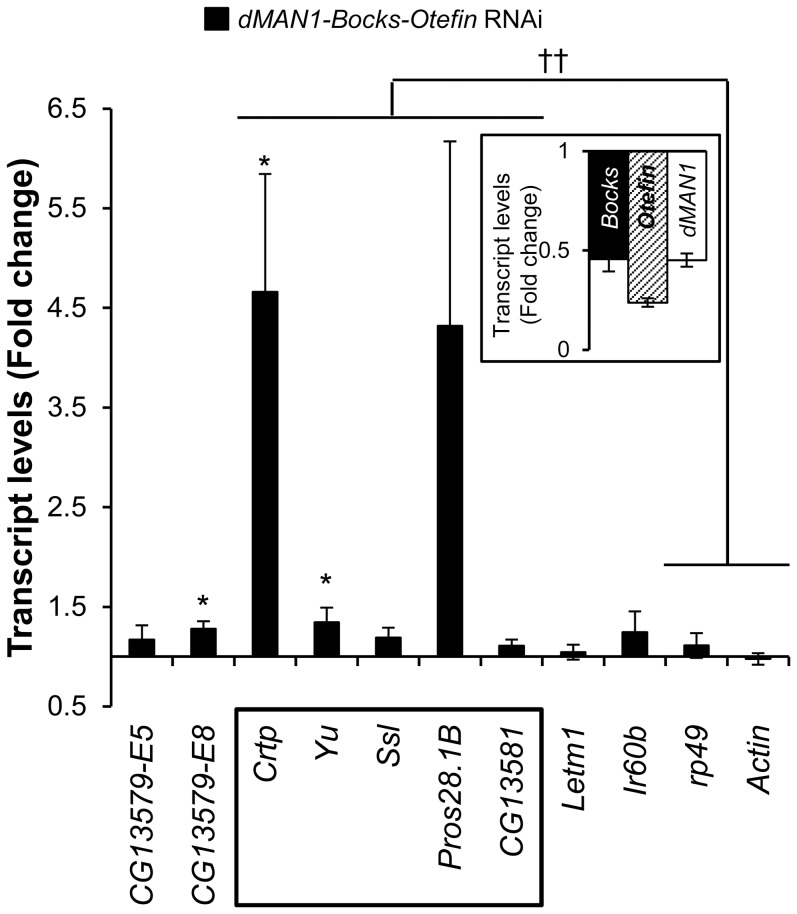
Effect of LEM domain protein depletion on the expression of the *60D1* gene-cluster. Bars show increased expression of the cluster genes in *S2* cells determined with RT-qPCR after treatment with the mixture of *dMAN1*, *Bocksbeutel*, and *Otefin* dsRNAs. The control *LacZ* dsRNA-treated cells served as the reference. Gene symbols are shown on the X-axis; the *60D1* cluster is boxed. n = 6; error bars show SEM; *, p≤0.05 for comparison of individual transcript levels between *LacZ* RNAi and target RNAi; ††, p≤0.01 for comparison between the *60D1* cluster and control housekeeping genes. Inserts show the knockdown efficiency of the RNAi at the RNA levels.

Because our findings indicated that HDAC1 is responsible for silencing and HDAC3 has an auxiliary role, the mechanisms that bring together these enzymes and the *60D1* locus warranted further inquiry. We hypothesized that HDAC/lamina-interacting proteins may be involved, and analyzed the role of lamina-associated “adaptors” (including LEM domain proteins and LBR) and the chromatin/lamin bridging factor, BAF, which have previously been shown to interact with HDACs [14], [15], [24], [25]. Our bioinformatics analysis of the gene expression database FlyAtlas [41] (http://flyatlas.org) identified Bocksbeutel, Otefin, and dMAN1 as three LEM domain proteins with appreciable expression in *S2* cells. The dsRNA approach was employed to downregulate these proteins individually or as a group in *S2* cells, in which derepression of the *60D1* cluster was analyzed by quantitative real-time PCR. An 80–90% efficient downregulation of Bocksbeutel, Otefin, or dMAN1 individually had no significant effect on expression of the *60D1* gene cluster, as compared to control *LacZ* dsRNA treatment (Figure S6). At the same time, simultaneous downregulation of all three LEM domain proteins caused a modest but significant up-regulation of the *60D1* cluster in contrast to control housekeeping genes (p = 0.009) (Figure 4A), even though the efficiency of downregulation of each LEM domain protein transcript in this case ranged between 50 and 75%. These findings indicate that LEM domain proteins probably contribute to repression of the *60D1* gene cluster and their roles appear redundant. Of note, functional redundancy between LEM domain proteins has been observed in unrelated genetic experiments [42] and therefore is not limited to our experimental system. A 60% downregulation of BAF did not cause significant derepression of the *60D1* cluster (Figure S6B) and a 95% efficient downregulation of LBR had only a minor effect on *Ssl*, one of the clustered genes (Figure S6B). These data provide little evidence for involvement of LBR and BAF in repression of the *60D1* gene cluster, even though the demonstrated interactions between BAF, LEM domain proteins, chromatin, and HDACs [15] make it tempting to suggest that BAF contributes to the LEM domain protein-mediated effect.

**Figure 5:**
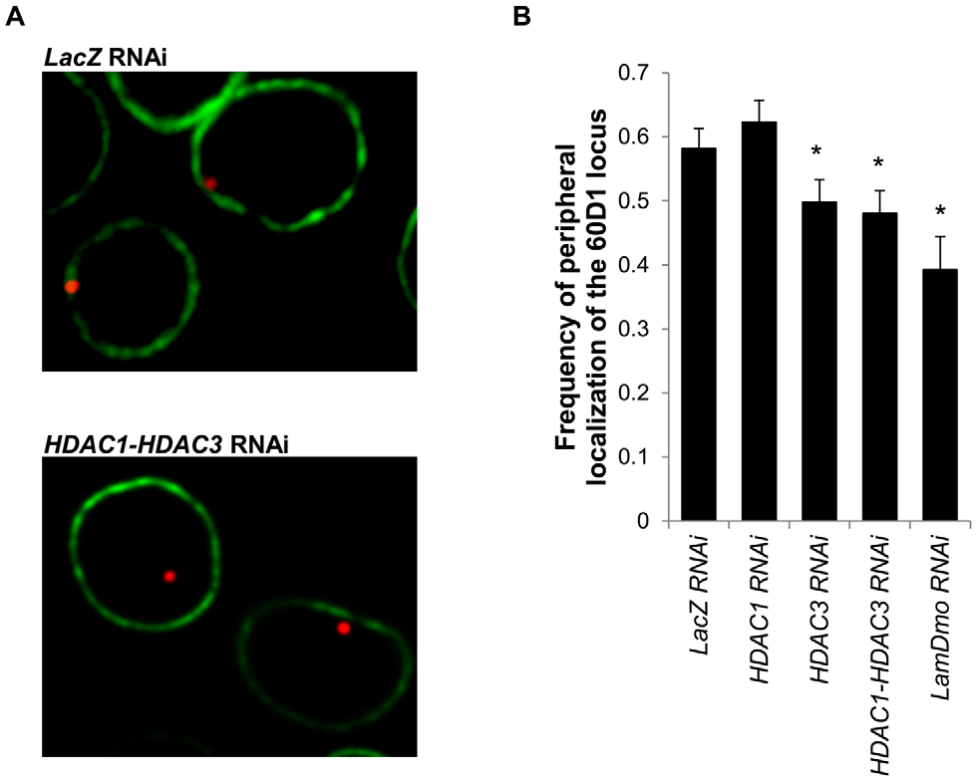
Effect of HDAC depletion on retention of the 60D1 locus at the nuclear periphery. (**A**) Position of the locus was determined by FISH (red) and the nuclear envelope visualized with immunostaining for *LamDm_o_* (green). Figure shows representative nuclei of cells treated with control *LacZ* dsRNA or a mixture of *HDAC1* and *HDAC3* dsRNAs. (**B**) Bars show the proportion of nuclei with FISH signals ≤0.4 µm apart from the nuclear envelope. dsRNAs used for depletion are indicated below the X-axis. *LacZ*, n = 256, 3 independent experiments; *HDAC1*, n = 199, 2 independent experiments; *HDAC3*, n = 201, 2 independent experiments; *HDAC1*+*HDAC3*, n = 208, 2 independent experiments; *LamDm_o_*, n = 89, 2 independent experiments. Error bars show SEM. *, p≤0.05 for comparisons to *LacZ* control.

Finally, we inquired into the role of HDACs and accessory proteins in retention of the *60D1* locus at nuclear lamina. Detachment of lamin-contacting chromatin from nuclear lamina has been observed in cells treated with a broad-range HDAC inhibitor, TSA [6], [43], raising the possibility that the role of HDACs in silencing of lamina-bound chromatin domains is limited to retention of these domains at the lamina. Using fluorescence *in situ* hybridization, we analyzed the localization of the *60D1* cluster in HDAC1/HDAC3-depleted *S2* cells and, as compared to control cells, observed a 17% reduction (p = 0.015) in frequency of nuclei in which the *60D1* cluster is in contact with nuclear lamina (i.e. the distance between the signal and the nuclear envelope is 0.4 µM or less) (Figures 5, S7). This reduction was not significantly different from the effect of depletion of B-type lamin *LamDm_o_* (p = 0.081 for this comparison) showing the major role of Class I HDACs in peripheral localization of the repressed locus. However, depletion of HDAC1 alone did not result in decreased frequency of nuclei with the *60D1* cluster in contact with the lamina (p = 0.19), indicating that this enzyme is required for silencing but not for positioning of the repressed domain at nuclear periphery. In contrast, depletion of HDAC3 resulted in a significant 15% reduction of the locus localization at the lamina (p = 0.036), demonstrating the key role of this enzyme in retention of the repressed domain at the lamina. Alternative approach to analysis of the FISH data by calculating the proportion of signals in peripheral 1/3 nuclear volume shell also showed that depletion of HDAC3 and HDAC1/3, but not HDAC1, reduces the peripheral localization of the locus (Figure S8).

In conclusion, our study provides direct experimental evidence for the long-persisting assumptions that HDACs are involved in gene silencing at the nuclear lamina, by identifying Class I enzymes HDAC1 and HDAC3 as the major players in this mechanism. We found that, likewise gene silencing, histone hypoacetylation and chromatin compaction in the multigenic chromatin domain are lamin-dependent. Moreover, we identified HDAC1 as the key factor required for silencing and specifically responsible for histone H4 deacetylation, and our data implicate HDAC3 as an auxiliary factor specifically responsible for localization of the repressed chromatin at the lamina. The “closed” chromatin configuration of the repressed domain also depends on HDAC1 and thus probably mediates the major repressory action of this enzyme at the nuclear periphery. Published data indicate that the *60D1* cluster interacts with HDAC1, in particular in the *Crtp* and *Pros28.1B* regions (Figure S9 and Table S6) [16], supporting direct involvement of this enzyme in histone deacetylation. We therefore propose a model in which Class I HDACs participate in lamina-dependent gene silencing through diverse pathways: HDAC1, tethered to the lamin scaffold by LEM domain proteins, is involved in deacetylation of histones H3 and H4 and “closing” of lamina-bound chromatin while HDAC3 contributes to histone H3 deacetylation and retention of the repressed chromatin at the lamina. Interestingly, a recent study showed that HDAC3 is also involved in peripheral localization of the lamina-interacting chromatin in mammals [44] indicating that this mechanism is conserved between diverse animals.

Lamina-associated chromatin domains harbor numerous cell type-specific genes that must be precisely regulated to orchestrate cell differentiation and development [45]–[47]. Genetic defects in the lamina components result in severe and currently incurable tissue degenerative disorders known as laminopathies [48]. Identification of the key role of Class I HDACs, and particularly HDAC1, in lamina-associated gene silencing implies that modulation of this enzyme may help to restore gene expression disrupted by nuclear lamina defects, and may be instrumental in establishing new expression patterns in pluripotent cells to guide their differentiation.

## Supporting Information

Figure S1 Efficiency of dsRNA-induced knockdowns. RNAi efficiency confirmed by Western blot for *LamDm_o_* and *HDAC1* dsRNA.
(TIF)

Figure S2 Effect of dsRNA on cell growth and cell toxicity. (**A**) A definite number of cells was seeded in multi-well plates and treated with dsRNA (n = 3 for each dsRNA). Cells were counted again at the end of the experiment. The growth rate was calculated as follow: Growth Rate = ln(N1/N0)/T, where N1 is the number of cells at the end of the experiment and N0 is the initial number of cells, T is the incubation time in hours. The graph represents growth rates shown as percentage with *LacZ* RNAi set to 100%. (**B**) Toxicity of dsRNA was evaluated with Live/Dead® Reduced Biohazard Viability/Cytotoxicity Kit. The graph represents the percent ratio of dead cells/total cells.
(TIF)

Figure S3 Location of the amplicons and evaluation of DNase I amount to be used for DNase I sensitivity assay. (**A**) Location of each amplicon along the *60D1* region is represented by an asterisk. Genes belonging to the testis-specific cluster are in black, surrounding genes are in grey. (**B**) Permeabilized *S2* cells were treated with different amount of DNase I (Control = no DNase I, U stands for unit). DNA damage at the locus *Actin5C* (active chromatin) and locus A21 (testis-specific locus, silent chromatin) was quantified by qPCR. Vertical axis shows the relative amount of DNA at each locus obtained after amplification; untreated control cells served as reference. n = 2; error bars show SEM; * or †, p≤0.05; **, p≤0.01 for comparisons to the control (* for Actin5C and † for A17). (**C**) Effect of LamDm_o_ RNAi on DNase I sensitivity of the chromatin at the level of the *Actin* and *RpL9* loci. Permeabilized cells were treated with DNase I and DNA damage was quantified by qPCR and normalized to the amplicons A37 and A39 (outside the *60D1* cluster). B-type lamin depletion does not result in increased sensitivity to DNase I digestion at the Actin and RpL9 loci. n = 3; error bars show SEM; *, p≤0.05.
(TIF)

Figure S4 Effect of Trichostatin A on transcript levels for the testis-specific cluster. Treatment of *S2* cells with 250 nM Trichostatin A for 48 hours leads to an increase in the expression of the testis-specific cluster *60D1*. Control cells were treated with vehicle (DMSO) and served as reference. n = 2 to 5 and the error bars represent SEM. Constitutively expressed transcript *Rp49* served as cDNA template loading reference. *Actin* = *Act5C*.
(TIF)

Figure S5 Effect of Class II, III and IV HDAC knockdowns on the expression of the testis-specific cluster. Cells were treated with dsRNAs specific for Class II HDACs (*HDAC2*; *HDAC4*), Class III HDAC (*dSIR2*) and Class IV HDAC (*HDACX*). Bars show levels of transcripts for the genes indicated below the X-axis (the *60D1* gene-cluster is framed). Control cells treated with *LacZ* dsRNA served as reference. n = 6; error bars show SEM; **, p≤0.01; ***, p≤0.001 for comparison between *LacZ* RNAi and target RNAi. *Rpl9* transcript was used as a template for loading control.
(TIF)

Figure S6 Effect of individual LEM domain protein knockdowns on the expression of the testis-specific cluster. (**A**) *S2* cells were incubated with dsRNA directed against *Bocksbeutel*, *Otefin*, or *dMAN1*; *LacZ* dsRNA–treated cells served as the reference. Transcript levels for the genes shown at bottom were determined by qRT-PCR. (**B**) *S2* cells were treated with *BAF* dsRNA and *LBR* dsRNA. Transcript levels for the genes shown at bottom were determined by qRT-PCR. The box outlines the genes comprising the *60D1* cluster. *Rpl9* transcript served as template for loading control. n = 6; error bars show SEM; *, p≤0.05; **, p≤0.01; ***, p≤0.001 for comparison between *LacZ* RNAi and target RNAi. Inserts show the knockdown efficiency of the RNAi at the RNA levels.
(TIF)

Figure S7 Raw image for Figure 5A. Figure shows representative nuclei of cells treated with control *LacZ* dsRNA.
(TIF)

Figure S8 Effect of class I HDAC RNAi on the positioning of the 60D1 locus within the nucleus. Graph obtained from data in Table S5. Nuclei were divided into three concentric spheres (0.126×R, 0.307×R and 1.000×R) representing equal volume. Bars show the ratio of number of nuclei with a FISH signal within the sphere interval (0.126×R) over total number of nuclei. dsRNAs used for depletion are indicated below the X-axis. *LacZ*, n = 106, n = 50 and n = 100; *HDAC1*, n = 49 and n = 150; *HDAC3*, n = 51 and n = 150; *HDAC1*+*HDAC3*, n = 108 and n = 100; *LamDm_o_*, n = 50 and n = 39. Error bars show SEM. *, p≤0.05 for comparisons to *LacZ* control.
(TIF)

Figure S9 Interaction of the *60D1* region with HDAC1. The graph represents DamID data from previous publication by Filion et al. [16] and shows the log ratio of signals obtained with HDAC1/Dam fusion over the control Dam experiment. Log ratios higher than 0 indicate interaction of HDAC1 with the corresponding genome region; positions of genes in the testis-specific cluster (outlined with a box) and beyond are shown.
(TIF)

Table S1 Primers used to obtain double strand RNAs.
(DOCX)

Table S2 Primers used to perform real-time PCR for expression studies.
(DOCX)

Table S3 Primers used to amplify genomic DNA after ChIP assay and DNaseI treatment.
(DOCX)

Table S4 FISH data analysis: ranked by the distance between FISH signal and lamina (D).
(XLSX)

Table S5 FISH data analysis: ranked by the ratio of distance between FISH signal and lamina/nuclear sphere radius (D/R).
(XLSX)

Table S6 Data used to generate the plot of HDAC1 enrichment across the *60D1* region.
(XLSX)
