# Supplementary figures and images for: Role of Histone Deacetylases in Gene Regulation at Nuclear Lamina
Source: PLoS One. 2012 Nov 30;7(11):e49692. doi: 10.1371/journal.pone.0049692 (PMC3511463; doi:10.1371/journal.pone.0049692)

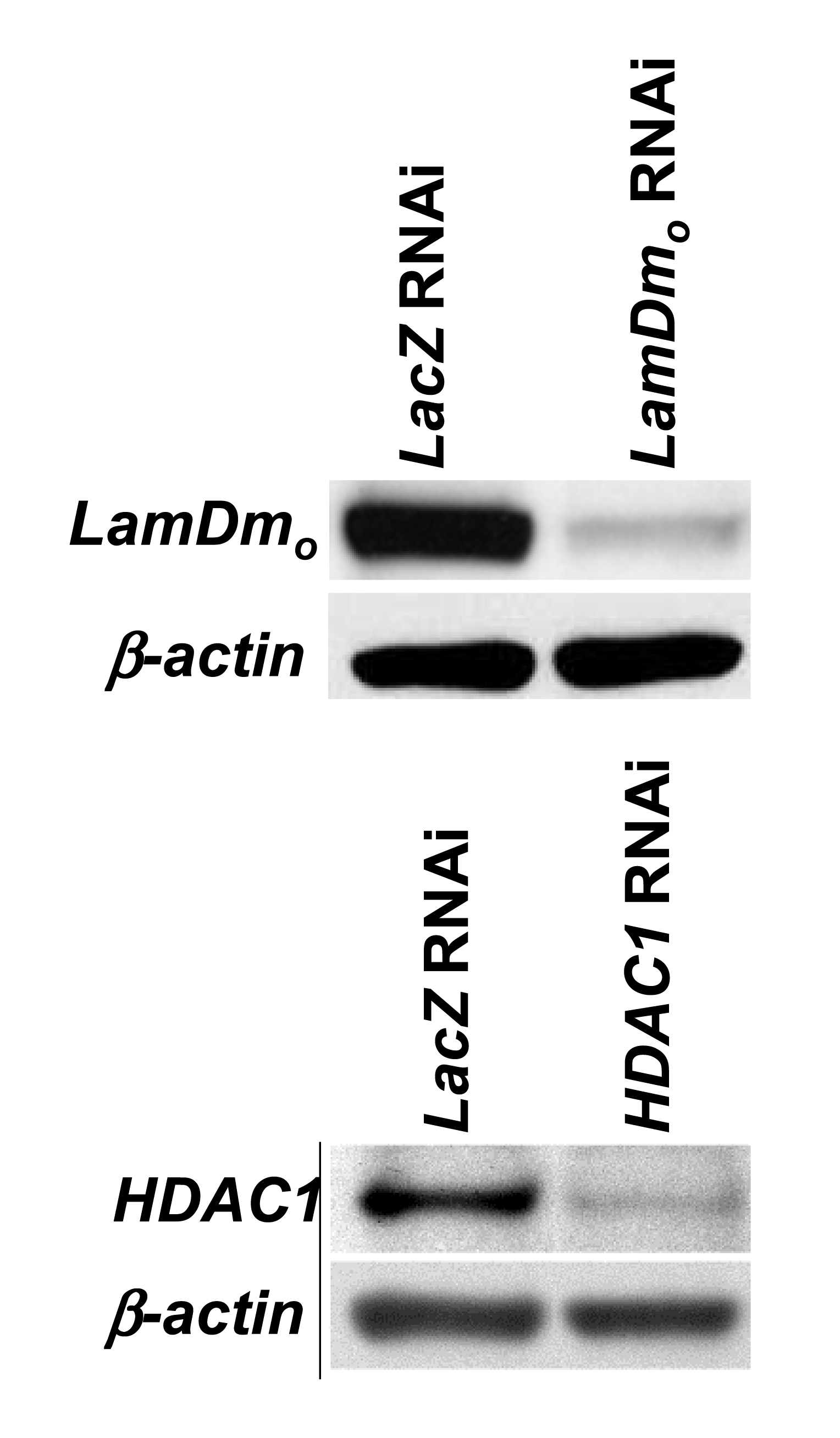

Supplement: Figure S1 — Efficiency of dsRNA-induced knockdowns. RNAi efficiency confirmed by Western blot for LamDmo and HDAC1 dsRNA. (TIF) [file pone.0049692.s001.tif]

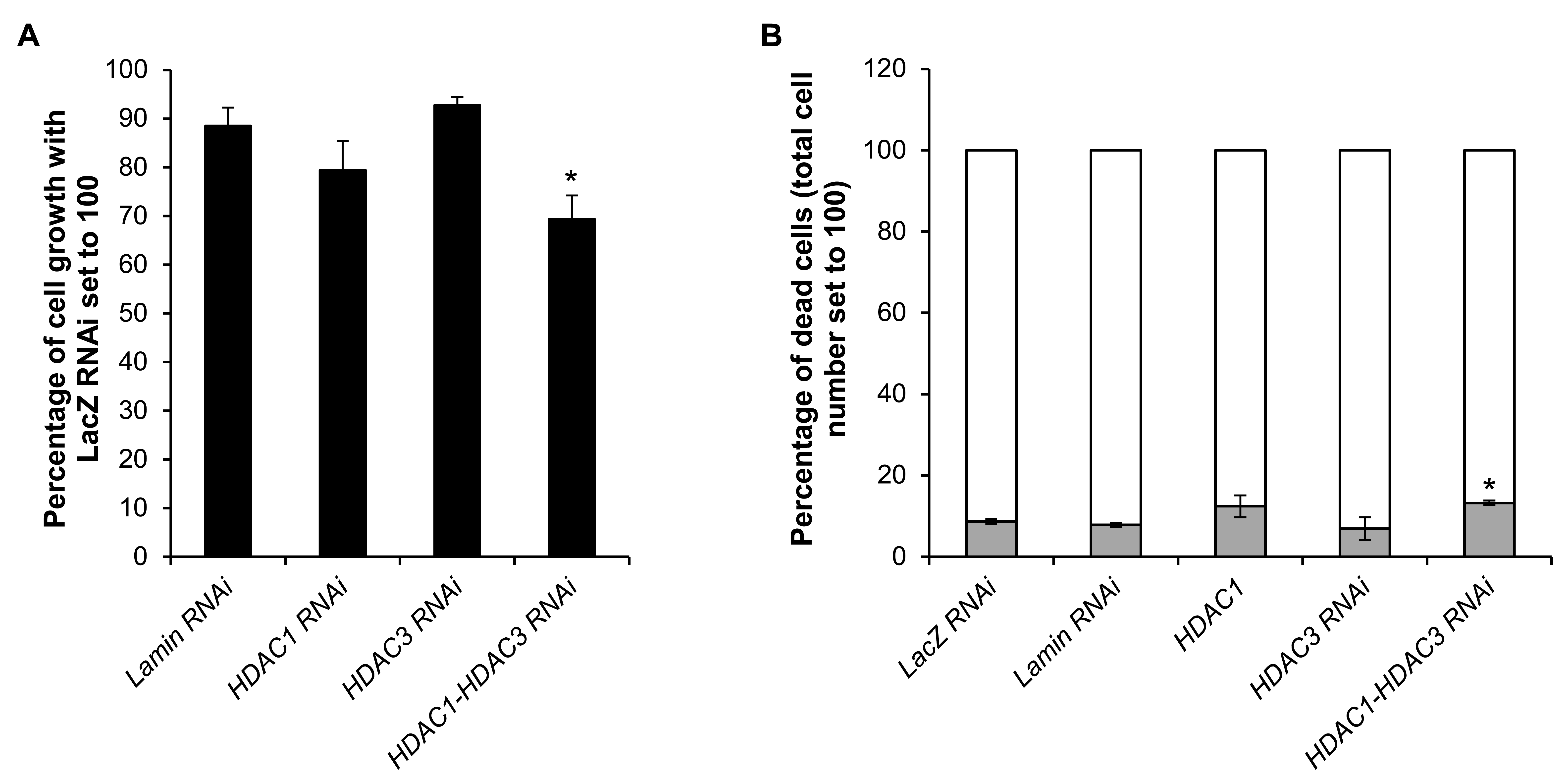

Supplement: Figure S2 — Effect of dsRNA on cell growth and cell toxicity. (A) A definite number of cells was seeded in multi-well plates and treated with dsRNA (n = 3 for each dsRNA). Cells were counted again at the end of the experiment. The growth rate was calculated as follow: Growth Rate = ln(N1/N0)/T, where N1 is the number of cells at the end of the experiment and N0 is the initial number of cells, T is the incubation time in hours. The graph represents growth rates shown as percentage with LacZ RNAi set to 100%. (B) Toxicity of dsRNA was evaluated with Live/Dead® Reduced Biohazard Viability/Cytotoxicity Kit. The graph represents the percent ratio of dead cells/total cells. (TIF) [file pone.0049692.s002.tif]

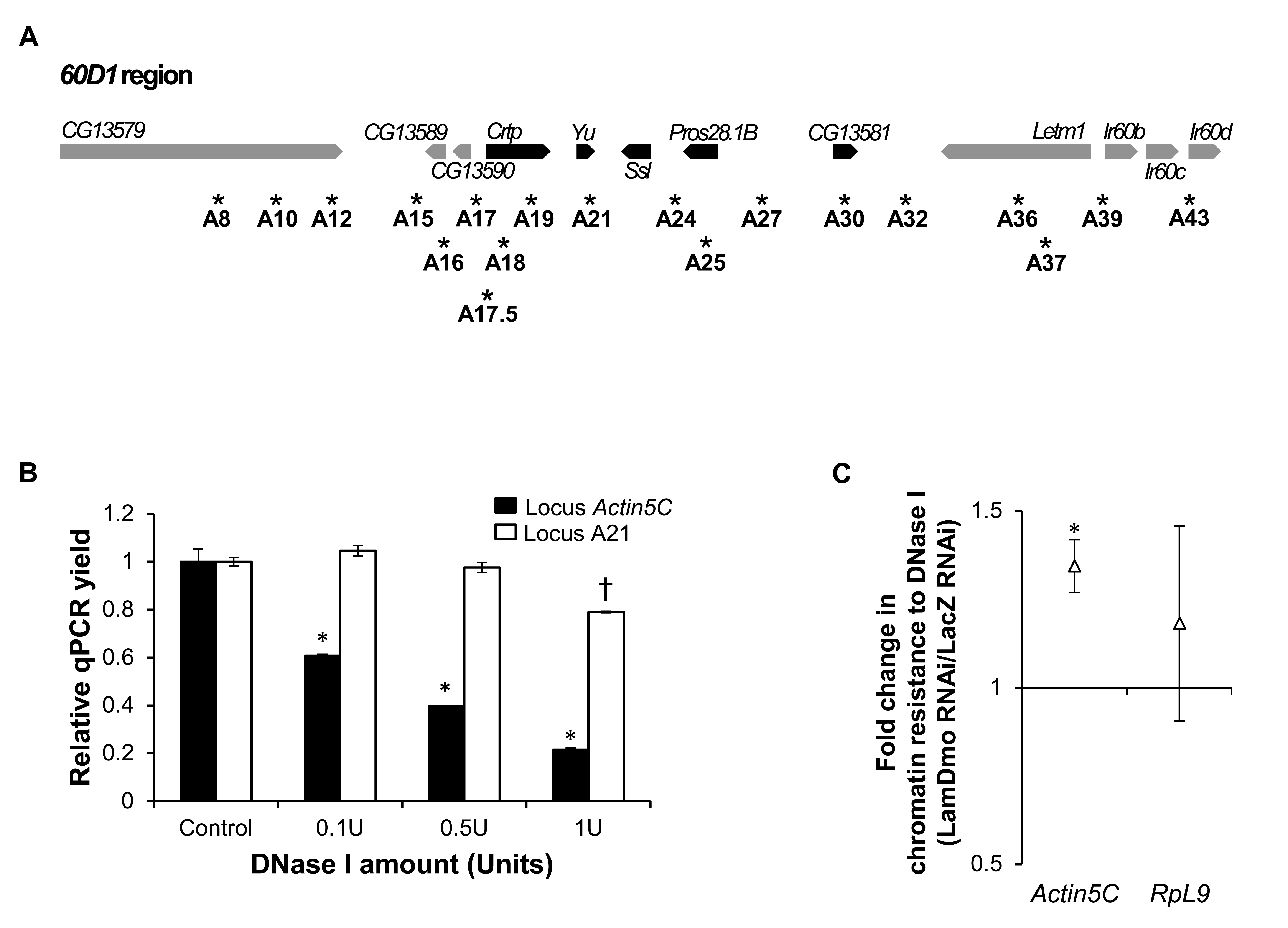

Supplement: Figure S3 — Location of the amplicons and evaluation of DNase I amount to be used for DNase I sensitivity assay. (A) Location of each amplicon along the 60D1 region is represented by an asterisk. Genes belonging to the testis-specific cluster are in black, surrounding genes are in grey. (B) Permeabilized S2 cells were treated with different amount of DNase I (Control = no DNase I, U stands for unit). DNA damage at the locus Actin5C (active chromatin) and locus A21 (testis-specific locus, silent chromatin) was quantified by qPCR. Vertical axis shows the relative amount of DNA at each locus obtained after amplification; untreated control cells served as reference. n = 2; error bars show SEM; * or †, p≤0.05; **, p≤0.01 for comparisons to the control (* for Actin5C and † for A17). (C) Effect of LamDmo RNAi on DNase I sensitivity of the chromatin at the level of the Actin and RpL9 loci. Permeabilized cells were treated with DNase I and DNA damage was quantified by qPCR and normalized to the amplicons A37 and A39 (outside the 60D1 cluster). B-type lamin depletion does not result in increased sensitivity to DNase I digestion at the Actin and RpL9 loci. n = 3; error bars show SEM; *, p≤0.05. (TIF) [file pone.0049692.s003.tif]

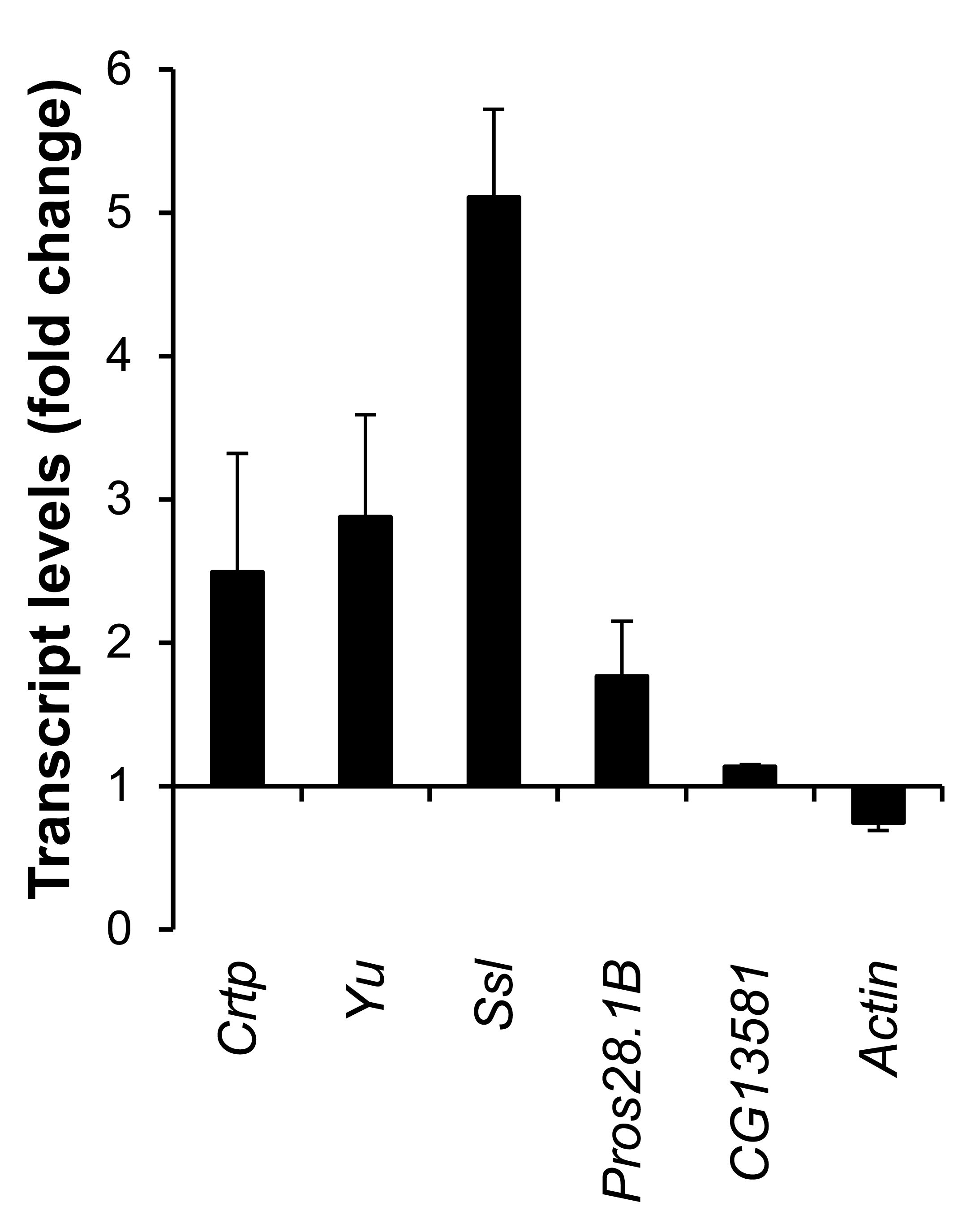

Supplement: Figure S4 — Effect of Trichostatin A on transcript levels for the testis-specific cluster. Treatment of S2 cells with 250 nM Trichostatin A for 48 hours leads to an increase in the expression of the testis-specific cluster 60D1. Control cells were treated with vehicle (DMSO) and served as reference. n = 2 to 5 and the error bars represent SEM. Constitutively expressed transcript Rp49 served as cDNA template loading reference. Actin = Act5C. (TIF) [file pone.0049692.s004.tif]

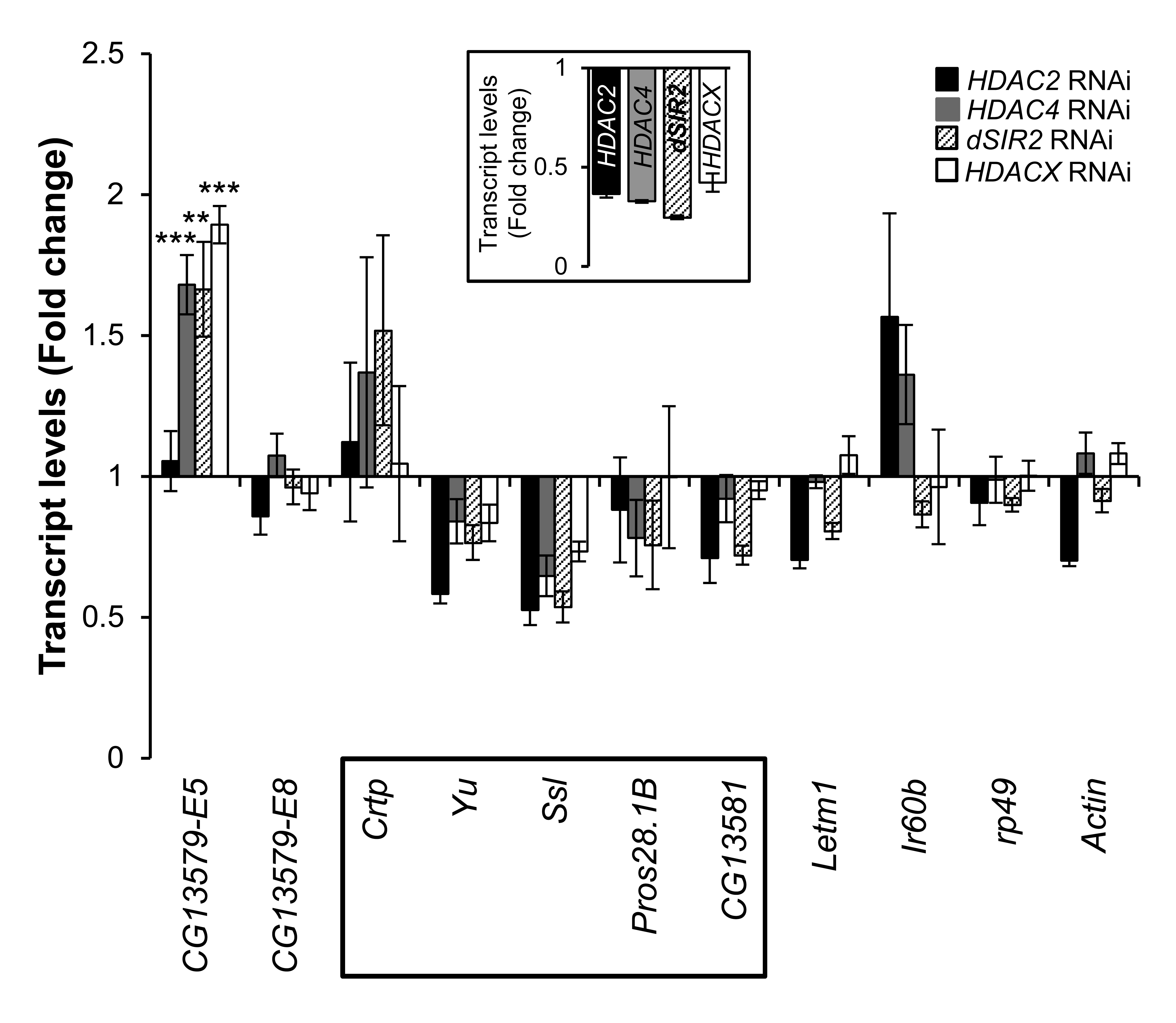

Supplement: Figure S5 — Effect of Class II, III and IV HDAC knockdowns on the expression of the testis-specific cluster. Cells were treated with dsRNAs specific for Class II HDACs (HDAC2; HDAC4), Class III HDAC (dSIR2) and Class IV HDAC (HDACX). Bars show levels of transcripts for the genes indicated below the X-axis (the 60D1 gene-cluster is framed). Control cells treated with LacZ dsRNA served as reference. n = 6; error bars show SEM; **, p≤0.01; ***, p≤0.001 for comparison between LacZ RNAi and target RNAi. Rpl9 transcript was used as a template for loading control. (TIF) [file pone.0049692.s005.tif]

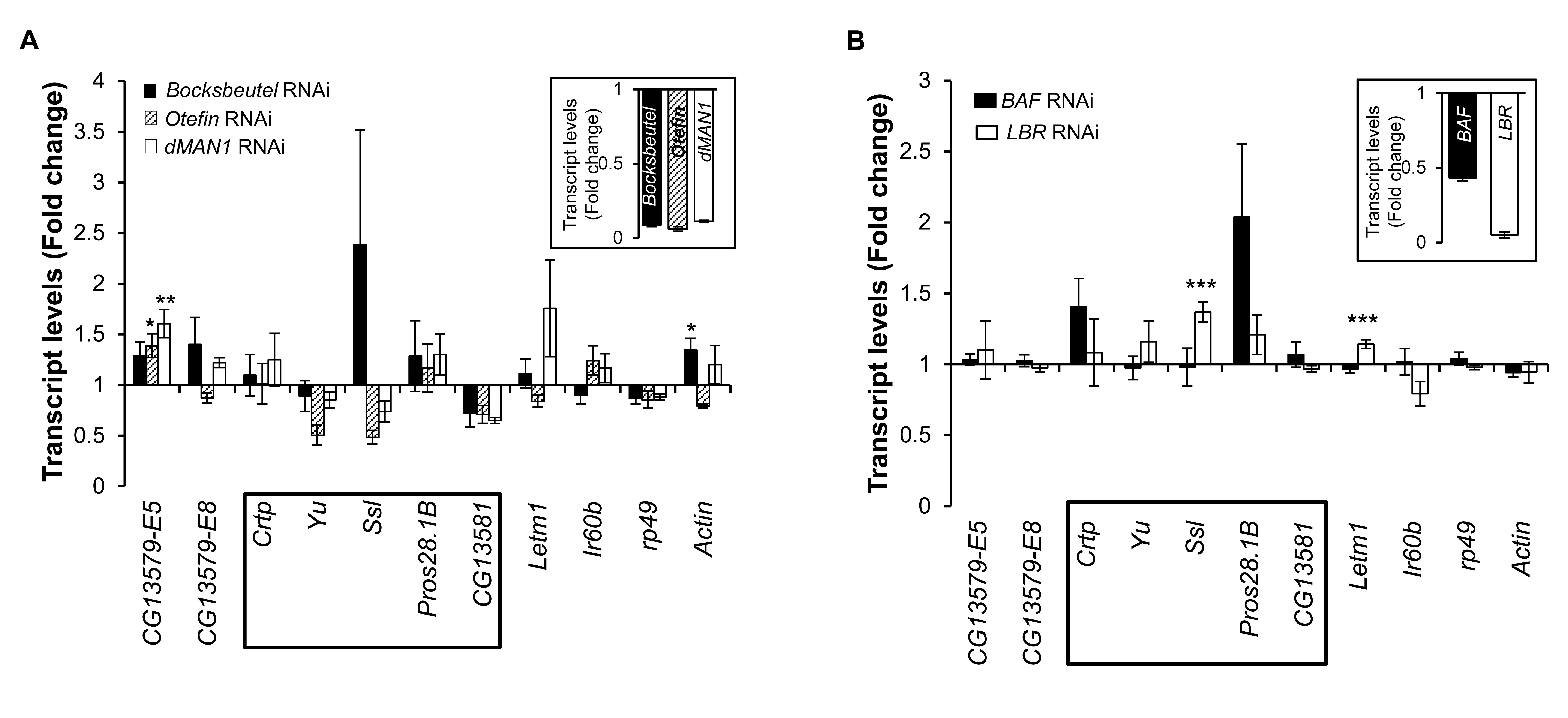

Supplement: Figure S6 — Effect of individual LEM domain protein knockdowns on the expression of the testis-specific cluster. (A) S2 cells were incubated with dsRNA directed against Bocksbeutel, Otefin, or dMAN1; LacZ dsRNA–treated cells served as the reference. Transcript levels for the genes shown at bottom were determined by qRT-PCR. (B) S2 cells were treated with BAF dsRNA and LBR dsRNA. Transcript levels for the genes shown at bottom were determined by qRT-PCR. The box outlines the genes comprising the 60D1 cluster. Rpl9 transcript served as template for loading control. n = 6; error bars show SEM; *, p≤0.05; **, p≤0.01; ***, p≤0.001 for comparison between LacZ RNAi and target RNAi. Inserts show the knockdown efficiency of the RNAi at the RNA levels. (TIF) [file pone.0049692.s006.tif]

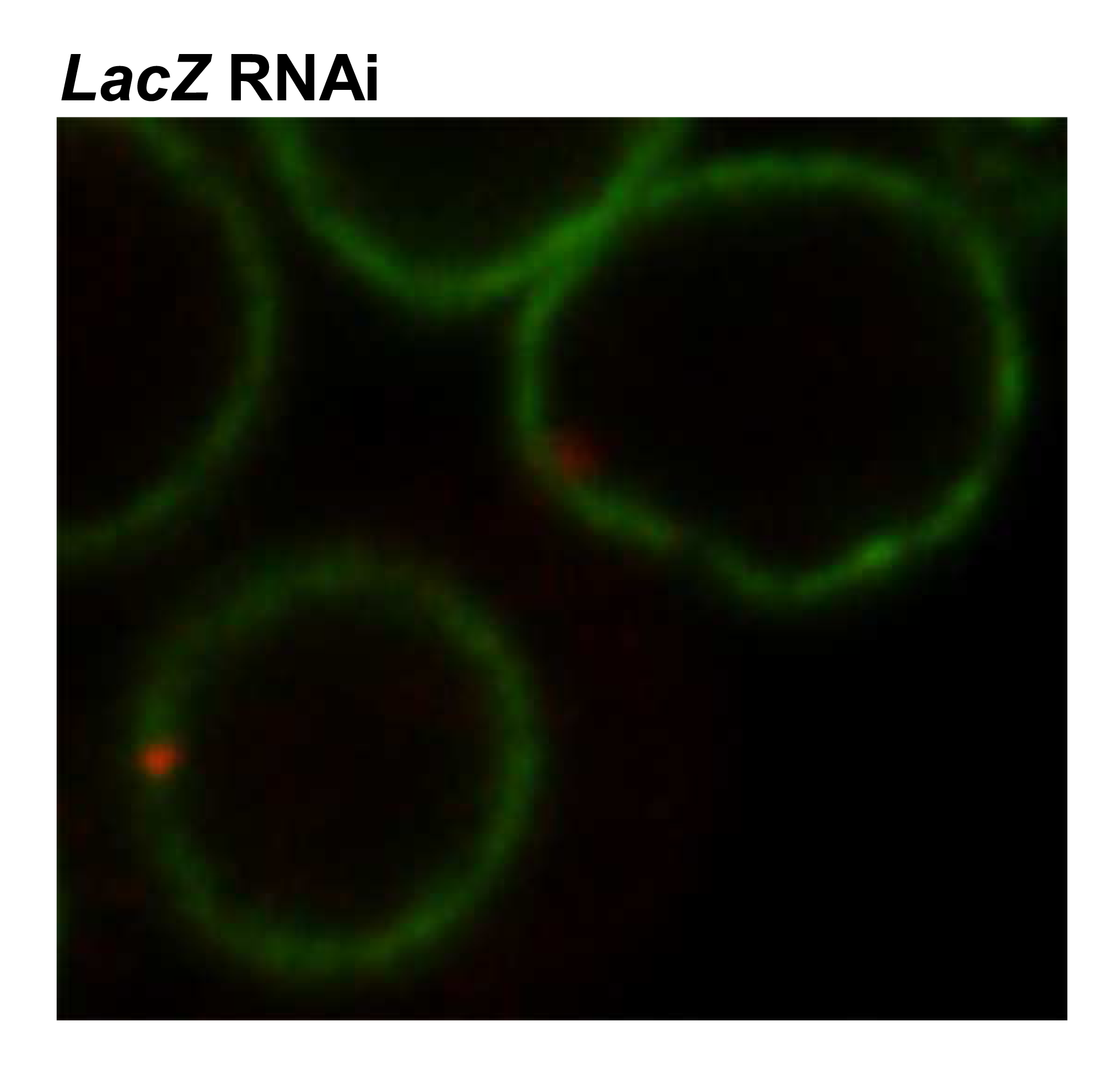

Supplement: Figure S7 — Raw image for Figure 5A. Figure shows representative nuclei of cells treated with control LacZ dsRNA. (TIF) [file pone.0049692.s007.tif]

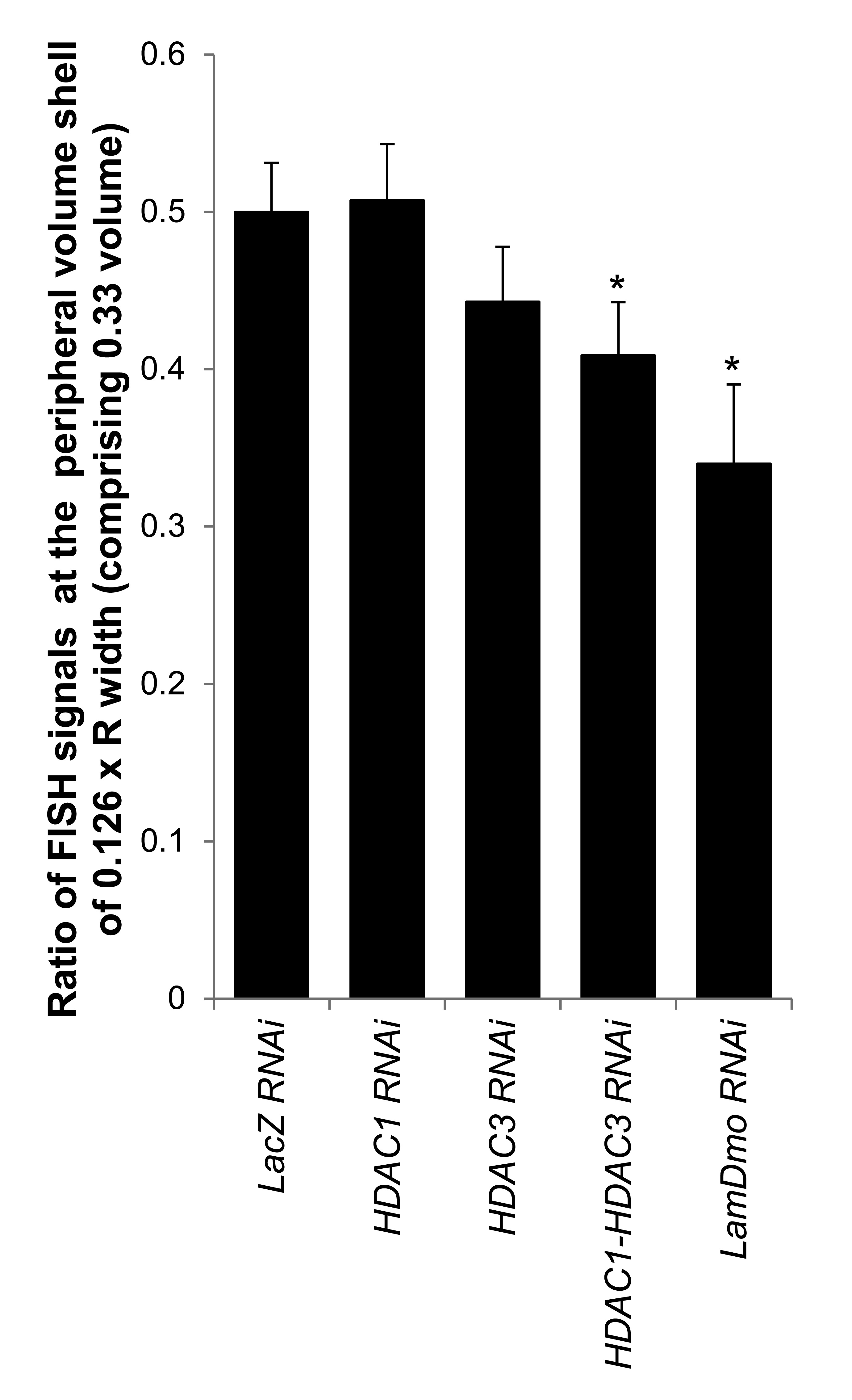

Supplement: Figure S8 — Effect of class I HDAC RNAi on the positioning of the 60D1 locus within the nucleus. Graph obtained from data in Table S5. Nuclei were divided into three concentric spheres (0.126×R, 0.307×R and 1.000×R) representing equal volume. Bars show the ratio of number of nuclei with a FISH signal within the sphere interval (0.126×R) over total number of nuclei. dsRNAs used for depletion are indicated below the X-axis. LacZ, n = 106, n = 50 and n = 100; HDAC1, n = 49 and n = 150; HDAC3, n = 51 and n = 150; HDAC1+HDAC3, n = 108 and n = 100; LamDmo, n = 50 and n = 39. Error bars show SEM. *, p≤0.05 for comparisons to LacZ control. (TIF) [file pone.0049692.s008.tif]

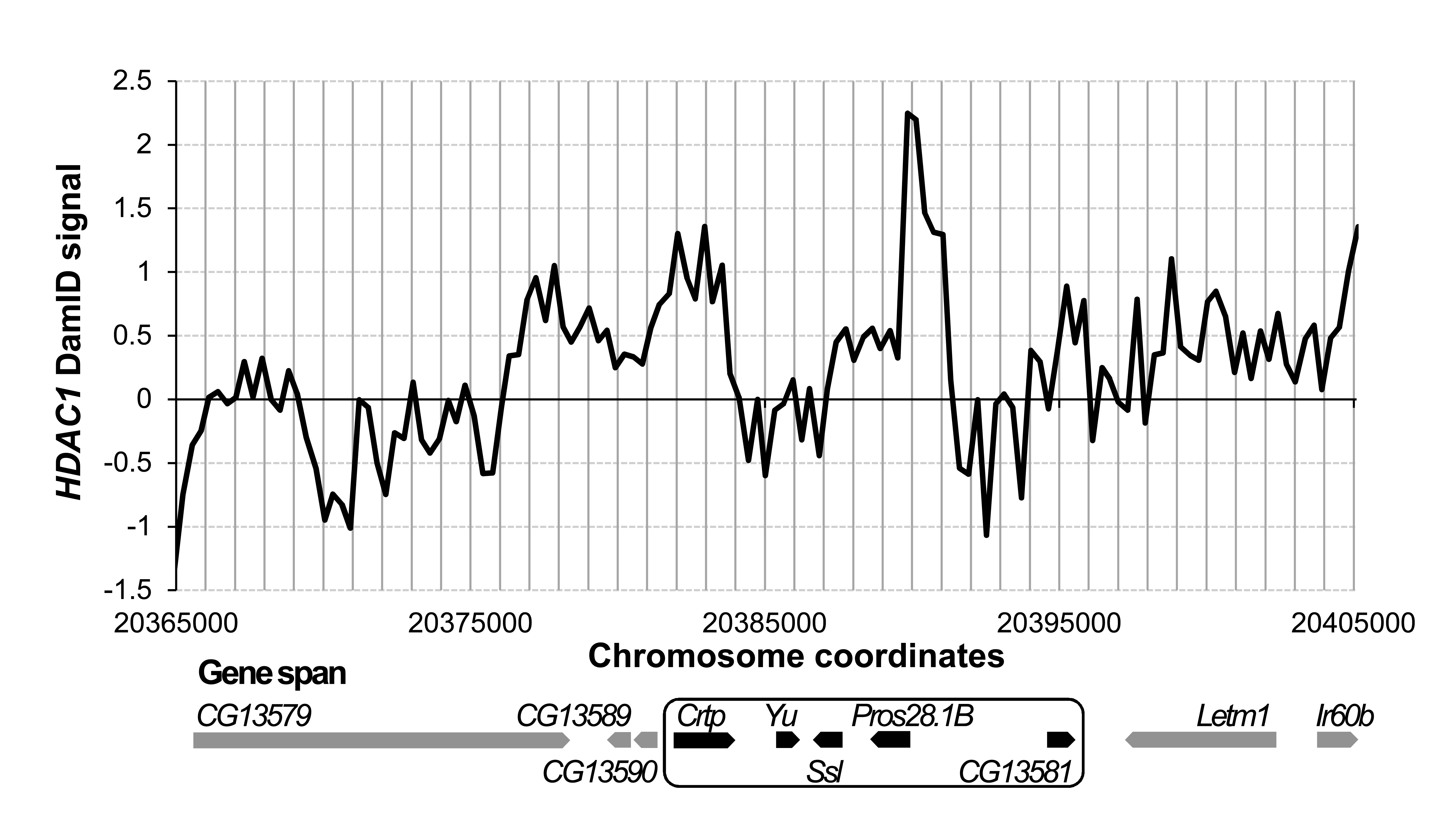

Supplement: Figure S9 — Interaction of the 60D1 region with HDAC1. The graph represents DamID data from previous publication by Filion et al. [16] and shows the log ratio of signals obtained with HDAC1/Dam fusion over the control Dam experiment. Log ratios higher than 0 indicate interaction of HDAC1 with the corresponding genome region; positions of genes in the testis-specific cluster (outlined with a box) and beyond are shown. (TIF) [file pone.0049692.s009.tif]
