# Supplementary material for: Role of Histone Deacetylases in Gene Regulation at Nuclear Lamina
Source: PLoS One. 2012 Nov 30;7(11):e49692. doi: 10.1371/journal.pone.0049692 (PMC3511463; doi:10.1371/journal.pone.0049692)
Supplement: Table S1 — Primers used to obtain double strand RNAs. (DOCX) [file pone.0049692.s010.docx]

**Table S1. Primers used to obtain double strand RNAs.**

|  | **Forward** | **Reverse** | |
| --- | --- | --- | --- |
| ***LacZ*** | gaattaatacgactcactatagggagaaacgtcgtgactgggaaaac | GAATTAATACGACTCACTATAGGGAGACCGCCACATATCCTGATCTT | |
| ***HDAC1*** | GGATCCTAATACGACTCACTATAGGGAGGATCCGTAGCTGCGGC | | GGATCCTAATACGACTCACTATAGGGAGGATGCCTTGTTGCTGT |
| ***HDAC2*** | GGATCCTAATACGACTCACTATAGGGAGGTCCCAAGGTGCTCTACATCAGCTT | | GATCCTAATACGACTCACTATAGGGAGGGCCATCCTCGCGCCACGCAC |
| ***HDAC3*** | GGATCCTAATACGACTCACTATAGGGAGGGAGCCCAGAAGCTGAAC | | GGATCCTAATACGACTCACTATAGGGAGGCGGGGTCTGCACCATTTGGACGCTGG |
| ***HDAC4*** | GGATCCTAATACGACTCACTATAGGGAGGCCCACGCACATCCACATCCACATG | | GGATCCTAATACGACTCACTATAGGGAGGTGACCTTGTGCGGCGGTGAA |
| ***HDACX*** | GGATCCTAATACGACTCACTATAGGGAGGGTTGCTCGTACAGAGGCGGT | | GGATCCTAATACGACTCACTATAGGGAGGGCCACCACTCAGCAGCATCAC |
| ***dSIR2*** | GGATCCTAATACGACTCACTATAGGGAGGACCCAGCAATCTTGTTCAGG | GGATCCTAATACGACTCACTATAGGGAGGCTGCTAACTGTCCTGGAGGC | |
| ***BAF*** | GAATTAATACGACTCACTATAGGGAGAGTGCGTCATTTTCAGAAACG | GAATTAATACGACTCACTATAGGGAGACAGCACCAGATACTGTCCCA | |
| ***Bocksbeutel*** | GAATTAATACGACTCACTATAGGGAGATCGGACCTTTCTTACCTGGAC | | GAATTAATACGACTCACTATAGGGAGATTGGCGAAATGGGTGAAAGTTTTG |
| ***Otefin*** | GAATTAATACGACTCACTATAGGGAGAATGGCCGATGTGGACGATTTTGAT | | GAATTAATACGACTCACTATAGGGAGAACCGGGTGCTGGGTTGCTCGCATA |
| ***LBR*** | GAATTAATACGACTCACTATAGGGAGACCCAGTCCAAGCAGCCCAGCC | | GAATTAATACGACTCACTATAGGGAGAGCAAAGGCACCCACCACTCGT |
| ***LamDm0*** | GAATTAATACGACTCACTATAGGGAGAATGTCGAGCAAATCCCGACG | | GAATTAATACGACTCACTATAGGGAGAGCGACTGCTTCAACTTGGCATC |
| ***dMAN1*** | GAATTAATACGACTCACTATAGGGAGACCGCTCAGGAAAGAAAGGCTA | | GAATTAATACGACTCACTATAGGGAGACACTTGAGCGGCAGATTGGGT |

HDAC1, 2, 3, 4 and X sequences are from reference [32]. Sequences for *Bocksbeutel*, *Otefin*, *Lamin B Receptor*, *LamDmo* and *dMAN1* are from references [36,S1]. Primers to obtain *LacZ*, *BAF* and *dSIR2* dsRNA were designed using the web-based tool SnapDragon (www.flyrnai.org/snapdragon_doc1.html). The underlined part of the primers represents a T7 RNA polymerase promoter sequence.

S1. Wagner N., Kagermeier B., Loserth S. Krohne G (2006) The Drosophila melanogaster LEM-domain protein MAN1. Eur.J.Cell Biol. 85: 91-105
