# Supplementary material for: Role of Histone Deacetylases in Gene Regulation at Nuclear Lamina
Source: PLoS One. 2012 Nov 30;7(11):e49692. doi: 10.1371/journal.pone.0049692 (PMC3511463; doi:10.1371/journal.pone.0049692)
Supplement: Table S2 — Primers used to perform real-time PCR for expression studies. (DOCX) [file pone.0049692.s011.docx]

**Table S2. Primers used to perform real-time PCR for expression studies.**

|  | **Forward** | **Reverse** |
| --- | --- | --- |
| ***HDAC1*** | GACCCACAACCTGCTGCTCA | CCGAATGGATCGCAAGAACC |
| ***HDAC2*** | AATTCTGCGCCTGCACACAG | CCAGCAGGGAGAGCTCGAAT |
| ***HDAC3*** | CATCCCATGAAGCCGCAAC | TAGGCCACGGAATTGCACTG |
| ***HDAC4*** | CGTTGTCGTTGTGCCATTCG | GGCAGGGATGGCATGAAACT |
| ***HDACX*** | CGCCTACATCCCTTCGATGC | TTGGTCAGCTCCGTGGGTTC |
| ***Sir2*** | AAGGAGCAGAGCGTGGATGC | GTGGCCATGACCGTGTGGTA |
| ***Bocksbeutel*** | TCGCCAGCGACAGAGTGTTC | GCGTCATTCGGCTGATCGTA |
| ***Otefin*** | GCCCGTATTCCAGACGCACT | TGCTCCAGGCTGTTCAACCA |
| ***dMAN1*** | CCACAACCGCCCCAAAACTA | CGGGCTTGAGTCGCTCTCAT |
| ***LBR*** | ATCCTGCCACCCTGTTCTCG | AGCAGCAAACAGCCGTAGCC |
| ***LamDm_o_*** | TTCGATGTACTTACGCTCCACCT | CTGGCTGGTTGTCACTTCCGTAG |
| ***CG13579-E5*** | CCCTTCTACAGCAAGCCATCCTACA | GGGCATGGTCGGATCGTTTAG |
| ***CG13579-E8*** | TTGAGGGCCTCTCCGAGAAGTACT | CTGCTGCGGCTGGTGGATCTG |
| ***Crtp*** | AGGCGGAGATTGCAGAACACGAG | CTGGGAGACACGGAGTGCCTGTA |
| ***Yu*** | AGCCTGCGGCGATACAATCA | ATGGCAATGTGGCTGGACTC |
| ***Ssl*** | CTTTGTTCGTGTGGAGTTAAAAATT | CTAGCTGTAACAACGTCTACATCCC |
| ***Pros28.1B*** | AGGGGTTCCACTGTGATGGGTCT | ATCGGGTTATGTACTCCACGGTC |
| ***CG13581*** | CGGTACGAACGCAGCCCAGACGA | TCATTGAAATTGGGCAGGACAGG |
| ***Letm1*** | CCACCATCCGGCGAATGAAG | TCGGGGATTTGGGGTCTGAC |
| ***Ir60b*** | CTCGCTGATCCTGCTGAATG | CGCGTTGTTCTCCCCTCTCA |
| ***Act5C*** | AGTTGCTGCTCTGGTTGTCG | CGTAGGACTTCTCCAACGAGG |
| ***Rpl9*** | CCACTGCCCAGAAGGACGAA | CTTGACGGTCGTGGACTGCT |
| ***Rp49*** | ATGACCATCCGCCCAGCATACAGG | GGTGCGCTTGTTCGATCCGTAACC |

Primers to assess for knockdown efficiency were designed using Primer3 [47]. Primers corresponding to the genes located in the region *60D1* (*Crtp, Yu, Ssl, Pros28.1B, CG13581, Letm1, Ir60b*) and the control genes (*Act5C*, *Rpl9, Rp49*) are from our previous publication [7].
