# Supplementary material for: Role of Histone Deacetylases in Gene Regulation at Nuclear Lamina
Source: PLoS One. 2012 Nov 30;7(11):e49692. doi: 10.1371/journal.pone.0049692 (PMC3511463; doi:10.1371/journal.pone.0049692)
Supplement: Table S3 — Primers used to amplify genomic DNA after ChIP assay and DNaseI treatment. (DOCX) [file pone.0049692.s012.docx]

**Table S3. Primers used to amplify genomic DNA after ChIP assay and DNaseI treatment.**

| **Primers for DNA amplification after ChIP assay and DNaseI treatment** | | |
| --- | --- | --- |
| **Amplicon** | **Forward** | **Reverse** |
| **A8** | AATGCTCGGCGGAACTCGGTAATG | CGGCGACTGCTGAAGTTGTAAGGA |
| **A10** | TCAATCGCCGCAAACATTCCCACA | CACAAAGCGGTCACACAATAATCC |
| **A12** | ACAGCAGACCACCTCCTTCAGCAA | ATCCTTGATCCCAGTGCCCTCTGT |
| **A15** | GCATATCATGTGCTGCTGTGGAAC | TTCGACGCAATGGTAGTTATTACA |
| **A16** | AAACTGACGGTGTACTGGTAAATAA | CTGCCATCTGGGGACTATCTACTAA |
| **A17** | CGGCTCCACGAACGTGACATTTAT | TTCCACTTCCGGCTATTCGATTTA |
| **A17.5** | ACCCTTGCCGAATCAACAACATTAC | CCGCTCGTCCGCCAACTTTGTGTCC |
| **A18** | GGATCGGGCTCAGTACAAGTGTCT | GCTTCCGCTCCAATTTCTGATACG |
| **A19** | TTTGAGCAGGCGGTCGAGATCATGG | GGCGACCGTAGGAGTATCCGTATAG |
| **A21** | CTTTCTGTATTTCGATGGGGTCTCT | CCGCGTCCTGTTACTGTTCCTGTTC |
| **A24** | GTGTAGGTGGAGCAGGAAATCGCAA | GTAATCAGCAGGAAAAATGTGTAAA |
| **A25** | CCTCCCATTGCTCTGCGTGTAGTT | GCCACCCGCACTTGCCTATTAGTT |
| **A27** | AACCCCCGTCGCCGTCCAAACTAA | TTCGAGGTCTGCCAACATTTTCAC |
| **A30** | CTTCTCCAGCTTCTTCTAATCTATC | TGTACTTGTCGTCTGGGCTGCGTTC |
| **A32** | TTCCCGTCCTGCCCATCCAGCATA | TCTTTGCCTGCCCGCTGACCCATA |
| **A36** | CACTCGCCAAAGGAGCTTAGAGCA | GTCACAGATGAACCCGCCAAAGAA |
| **A37** | GCAGGTGCCTCTTTGTCTGGTCTT | GTGTGTGTTAGCGGGCATTTTGTT |
| **A39** | ACTTTTCCGGCAGTTATACGACTT | CGTGTCGCGCATATCCTCGAGATT |
| **A43** | TTAAAAATGCGGCTTGCCATATAC | TTTATAACGCGGAATGTTGGGAAT |
| **Actin5C** | AGTTGCTGCTCTGGTTGTCG | CGTAGGACTTCTCCAACGAGG |
| **RpL9** | CCACTGCCCAGAAGGACGAA | CTTGACGGTCGTGGACTGCT |

Primer sequences are from our previous publication [34].
